# Comparison of intrathecal morphine with continuous patient-controlled epidural anesthesia versus intrathecal morphine alone for post-cesarean section analgesia: a randomized controlled trial

**DOI:** 10.1186/s12871-020-01050-6

**Published:** 2020-06-03

**Authors:** Izumi Sato, Hajime Iwasaki, Sarah Kyuragi Luthe, Takafumi Iida, Hirotsugu Kanda

**Affiliations:** 1 Department of Anesthesiology and Critical Care Medicine, Asahikawa Medical University, Midorigaoka-higashi 2-1-1-1, Asahikawa, Hokkaido 078-8510 Japan; 2 Department of Anesthesiology, Indiana University School of Medicine, 1130 W. Michigan Street, Fesler Hall 204, Indianapolis, IN 46202 USA

**Keywords:** Cesarean section, Postoperative analgesia, Intrathecal morphine, Patient-controlled epidural analgesia

## Abstract

**Background:**

Several neuraxial techniques have demonstrated effective post-cesarean section analgesia. According to previous reports, it is likely that patient-controlled epidural analgesia (PCEA) without opioids is inferior to intrathecal morphine (IM) alone for post-cesarean section analgesia. However, little is known whether adding PCEA to IM is effective or not. The aim of this study was to compare post-cesarean section analgesia between IM with PCEA and IM alone.

**Methods:**

Fifty patients undergoing elective cesarean section were enrolled in this prospective randomized study. Patients were randomized to one of two groups: IM group and IM + PCEA group. All patients received spinal anesthesia with 12 mg of 0.5% hyperbaric bupivacaine, 10 μg of fentanyl, and 150 μg of morphine. Patients in IM + PCEA group received epidural catheterization through Th11–12 or Th12-L1 before spinal anesthesia and PCEA (basal 0.167% levobupivacaine infusion rate of 6 mL/h, bolus dose of 3 mL in lockout interval of 30 min) was commenced at the end of surgery. A numerical rating scale (NRS) at rest and on movement at 4,8,12,24,48 h after the intrathecal administration of morphine were recorded. In addition, we recorded the incidence of delayed ambulation and the number of patients who requested rescue analgesics. We examined NRS using Bonferroni’s multiple comparison test following repeated measures analysis of variance; *p < 0.05* was considered as statistically significant.

**Results:**

Twenty-three patients in each group were finally analyzed. Mean NRS at rest was significantly higher in IM group than in IM + PCEA group at 4 (2.7 vs 0.6), 8 (2.2 vs 0.6), and 12 h (2.5 vs 0.7), and NRS during mobilization was significantly higher in IM group than in IM + PCEA group at 4 (4.9 vs 1.5), 8 (4.8 vs 1.9), 12 (4.9 vs 2), and 24 h (5.7 vs 3.5). The number of patients who required rescue analgesics during the first 24 h was significantly higher in IM group compared to IM + PCEA group. No significant difference was observed between the groups in incidence of delayed ambulation.

**Conclusions:**

The combined use of PCEA with IM provided better post-cesarean section analgesia compared to IM alone.

**Trial registration:**

UMIN-CTR (Registration No. UMIN000032475). Registered 6 May 2018 – Retrospectively registered.

## Background

Several neuraxial techniques have demonstrated effective postoperative analgesia following cesarean section [1–4]. Intrathecal or epidural morphine and patient-controlled epidural anesthesia (PCEA) are generally used for post-cesarean section analgesia. One study reported that intrathecal morphine alone was superior to epidural morphine alone or PCEA without opioids for postoperative analgesia following cesarean section [1]. Both intrathecal and epidural morphine are reported to be effective for post-cesarean section analgesia [5, 6], however, it is unknown if there is a meaningful difference between the route through which a single dose of neuraxial morphine is administered. Another study concluded that the combined use of intrathecal morphine and PCEA improved post-cesarean section analgesia compared to PCEA without opioids [2]. Based on the literature and one retrospective study [7], it is likely that PCEA without opioids is inferior to intrathecal morphine alone for post-cesarean section analgesia. In other words, performing PCEA without opioids may not be a reason to omit intrathecal morphine. However, little is known whether adding epidural anesthesia to intrathecal morphine is effective or not. We hypothesized that the combined use of PCEA and intrathecal morphine may have an advantage in post-cesarean section analgesia compared to intrathecal morphine alone.

## Methods

This study was registered in the University Hospital Medical Information Network under registration number UMIN000032475 with approval from the hospital’s ethics committee. This study adheres the applicable CONSORT guidelines. Healthy pregnant women scheduled for cesarean section at Kushiro Red Cross Hospital (Hokkaido, Japan) were enrolled in this study. Written informed consent was obtained from all the patients. We included patients of the American Society of Anesthesiologists physical status classification scale I and II. We excluded patients with contraindications for spinal or epidural anesthesia due to hemodynamic, infectious, hemostatic, neurological statuses, and medication use. In addition, we excluded cases of which we were unable to obtain informed consent such as extremely emergent cesarean sections, and cases of which general anesthesia was selected for reasons such as urgency or predicted massive hemorrhage. Using sealed envelopes, patients were randomly divided into two groups: Group IM (intrathecal morphine alone) and Group IM + PCEA (intrathecal morphine combined with PCEA).

Patients in the IM + PCEA group received epidural catheterization prior to spinal anesthesia. A 19-gauge epidural catheter with an 18-gauge epidural Tuohy needle was inserted 5 cm through the Th11–12 or Th12-L1 vertebral interspace. All patients received spinal anesthesia at the L2–3 or L3–4 vertebral interspace with a 25-gauge Quincke spinal needle (TOP Corp., Tokyo, Japan) with 0.5% hyperbaric bupivacaine (12 mg), fentanyl (10 mcg), and morphine (150 mcg) administered. Prior to spinal anesthesia, rapid infusion of 6% hydroxyethyl starch 130/0.4 (Voluven, Fresenius Kabi Japan, Tokyo, Japan) and a total of 1000 ml was administered during surgery. Systolic blood pressure was maintained above 100 mmHg using boluses of phenylephrine 100mcg. A bolus of droperidol 1.25 mg was administered to treat intraoperative nausea and vomiting when necessary. In the IM + PCEA group, continuous epidural infusion of 0.167% levobupivacaine using disposable PCEA infusers (Smiths Medical Japan, Tokyo, Japan) were commenced at the end of surgery and ceased after 24 h. The PCEA settings were basal infusion rate of 6 mL/h, patient-controlled analgesia (PCA) demand dose of 3 mL, and lockout interval of 30 min. To confirm the effect of PCEA, cold sensory blockade was assessed prior to removal of the epidural catheter. We excluded patients with insufficient or unilateral sensory block from the analysis. In the IM + PCEA group, the epidural catheter was removed 24 h after intrathecal administration of morphine but prior to ambulation. All patients began ambulation 24 h after intrathecal administration of morphine. Oxygen saturation was monitored for 24 h after surgery for concerns of respiratory depression potentially related to morphine.

We recorded postoperative pain scores using an 11-point verbal score numerical rating scale (NRS) ranging from 0 as no pain to 10 as worst imaginable pain, at rest and on movement (sitting in an upright position and movement of lower extremities) at 4, 8, 12, 24, 48 h after intrathecal administration of morphine. In addition, we assessed the intensity of motor blockade of lower extremities according to the Bromage score [8] (score 1 = free movement of legs and feet; score 2 = just able to flex knees with free movement of feet; score 3 = unable to flex knees, but with free movement of feet; and score 4 = unable to move legs or feet). Inadequate analgesia was managed with 50 mg diclofenac suppository or a drip infusion of 50 mg flurbiprofen axetil for the first 24 h. Morphine-induced side effects including pruritus and postoperative nausea and vomiting (PONV) were treated with 25 mg of hydroxyzine hydrochloride drip infusion and 10 mg of intravenous metoclopramide infusion, respectively. All data were collected by an investigator who was not involved in providing anesthesia. In addition, patients were asked to rate their satisfaction with analgesia before discharge as follows; 5:completely satisfied, 4:satisfied, 3:fair, 2:unsatisfied, 1:completely unsatisfied.

The primary outcome of this study was postoperative pain as measured by NRS at 12 h after intrathecal administration of morphine during mobilization. Secondary outcomes were NRS at 12 h after intrathecal administration of morphine at rest, NRS and Bromage score at 4, 8, 24, 48 h after intrathecal administration of morphine at rest and during mobilization, the number of patients who requested rescue analgesics, the number of requests for rescue analgesics per patient, the interval time before the first request of rescue analgesics, the incidence of delayed ambulation, the incidence of requested treatment for pruritus and PONV during the first 24 h after intrathecal administration of morphine, and patient satisfaction before discharge.

### Statistical analysis

The sample size calculation of 21 patients for each group to provide an α value of 0.05 and a β value of 0.1, was based on NRS of 10 previous post-cesarean section patients who were not included in the final analysis (5 patients who received intrathecal morphine and 5 patients who received both intrathecal morphine and PCEA) during mobilization at 12 h after intrathecal administration of morphine. We adjusted our sample size of 25 patients for each group for anticipated dropouts.

Results are expressed as mean ± standard deviation (SD), unless stated otherwise. We examined NRS and Bromage scores using Bonferroni’s multiple comparison test following repeated measures analysis of variance. Differences between groups were compared using unpaired t-test for patient characteristics and analgesic satisfaction, and Mann-Whitney U test for rescue analgesics and morphine-induced side effects. For categorical data, we used Fisher’s exact test. All statistical analyses were performed using GraphPad Prism® version 7.03 (GraphPad Software, Inc., La Jolla, CA) and a *P*-value of < 0.05 was considered statistically significant.

## Results

**Fig. 1:**
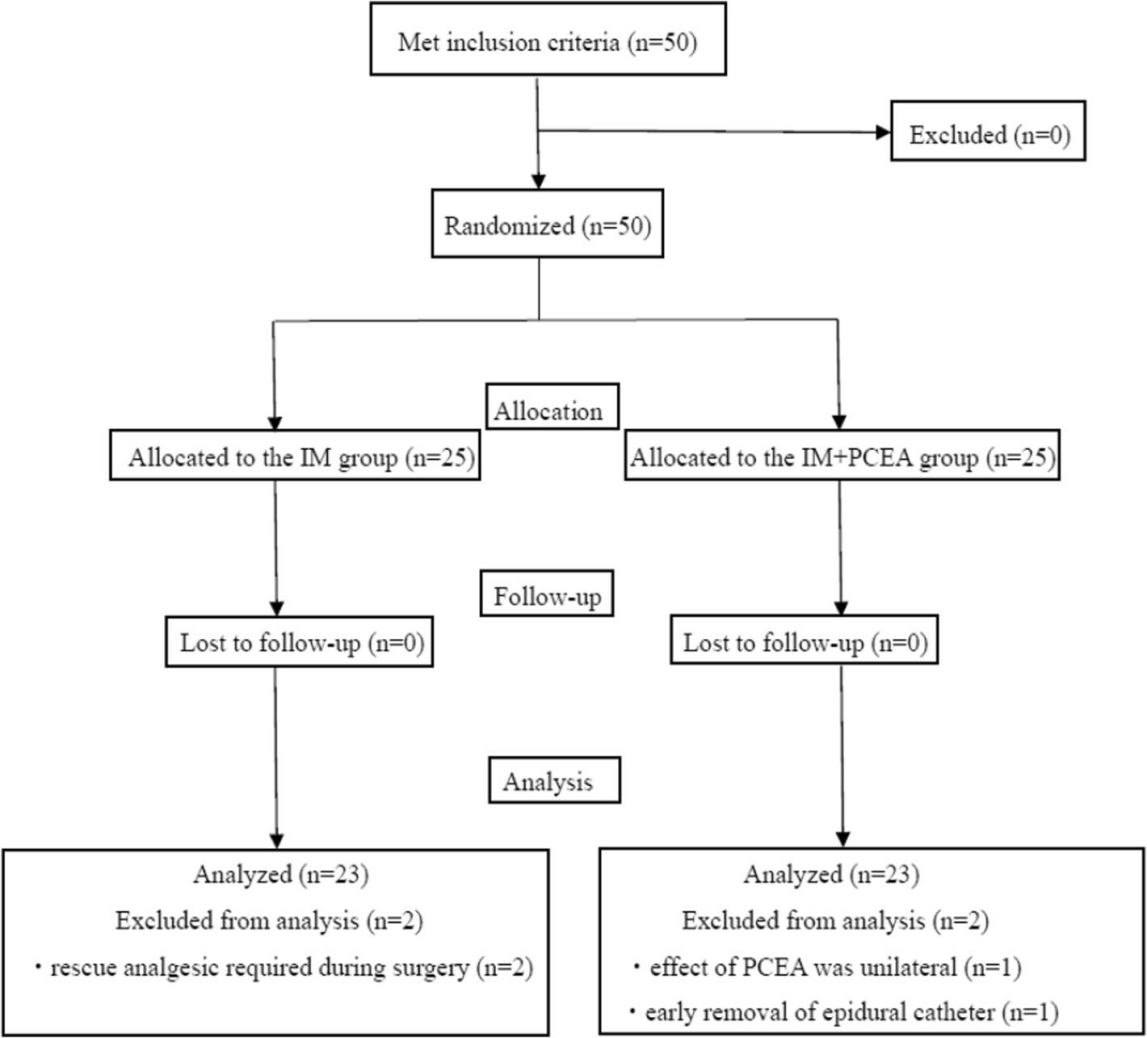
CONSORT diagram

Fifty pregnant women (aged 20–45 years) scheduled for cesarean section were enrolled in this study between January 2017 and April 2018. The CONSORT diagram is showed in Fig. 1. We excluded 2 patients in IM group due to use of rescue analgesic during the surgery and 2 patients in IM + PCEA group due to insufficient effect of PCEA and early removal of epidural catheter. Finally, 23 patients in each group were analyzed. Patient characteristics and intraoperative data were comparable among the groups (Table 1). Twelve patients in the IM group and 11 patients in the IM + PCEA group used antiemetic drugs during surgery.

**Table 1:** Patient characteristics and intraoperative data

	IM group (*n* = 23)	IM + PCEA group (*n* = 23)	*P* value
Age (years)	33.30 ± 5.46	32.74 ± 4.98	0.7155
Height (cm)	157.87 ± 6.27	158.00 ± 5.89	0.9423
Weight (kg)	65.48 ± 10.20	64.65 ± 7.84	0.7596
Duration of surgery (minutes)	53.48 ± 10.30	51.13 ± 9.60	0.4281
Previous history of caesarean section	13 (56.5)	14 (60.9)	> 0.9999
ASA physical status I/II	12 (52.2)/11 (47.8)	16 (69.6)/7 (30.4)	0.3651

Results are expressed as mean ± SD or as n (%). *IM* Intrathecal morphine, *IM + PCEA* Intrathecal morphine combined with patient-controlled epidural anesthesia, *ASA* American Society of Anesthesiologists

**Fig. 2:**
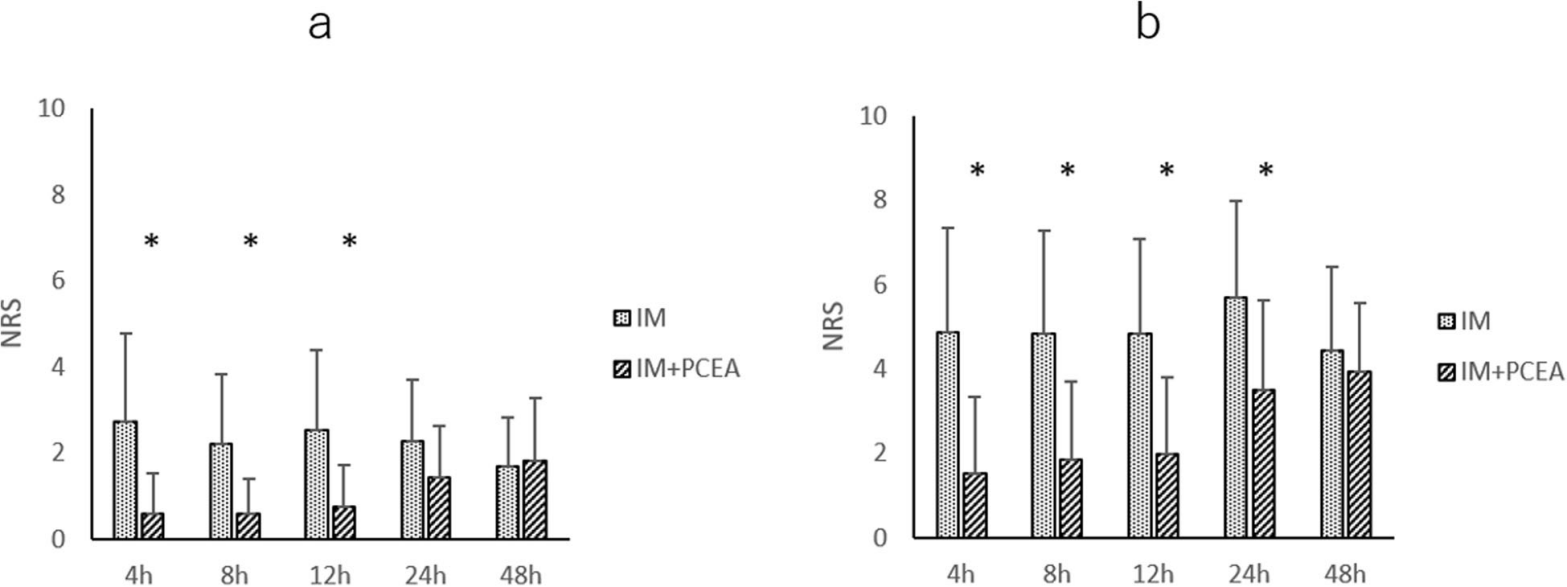
Numerical rating scale (NRS) during the first 48 h after intrathecal administration of morphine at rest and at movement. **a**. NRS at rest for the first 48 h after intrathecal administration of morphine. **b**. NRS at movement for the first 48 h after intrathecal administration of morphine. *P<0.01. IM = intrathecal morphine. IM + PCEA = intrathecal morphine combined with patient-controlled epidural anesthesia

NRS obtained during the first 48 h are shown in Fig. 2. Mean NRS at rest (Fig. 2a) was significantly higher in IM group than in IM + PCEA group at 4 (2.7 vs 0.6), 8 (2.2 vs 0.6), and 12 h (2.5 vs 0.7), and NRS during mobilization (Fig. 2b) was significantly higher in IM group than in IM + PCEA group at 4 (4.9 vs 1.5), 8 (4.8 vs 1.9), 12 (4.9 vs 2), and 24 h (5.7 vs 3.5). In IM + PCEA group, 6 out of 23 patients (26.1%) used PCA after surgery, and the frequency of use of PCA was 0.78 ± 1.86 (mean ± SD).

**Fig. 3:**
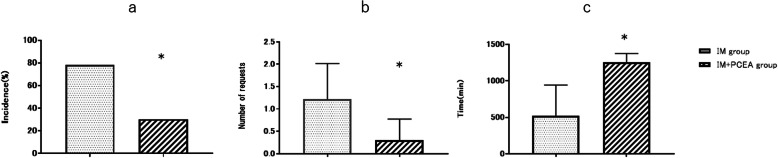
Requests for rescue analgesics during the first 24 h after intrathecal administration of morphine. **a**. Percentage of patients who requested rescue analgesics. **b**. Number of requests for rescue analgesics per patient. *P*<0.0001. **c**. Interval time (minutes) before the first request for rescue analgesics. *P* = 0.0005. IM = intrathecal morphine. IM + PCEA = intrathecal morphine combined with patient-controlled epidural anesthesia

With respect to requests for rescue analgesics, significant differences were observed among the two groups (Fig. 3). The number of patients who required rescue analgesics during the first 24 h was 18 (78.3%) in IM group, and 7 (30.4%) in IM + PCEA group (Fig. 3a). The number of requests for rescue analgesics per patient was also significantly higher in IM group (1.22 ± 0.80) than in IM + PCEA group (0.3 ± 0.47) (Fig. 3b). The interval time before the first request for rescue analgesics in IM + PCEA group (1254 ± 120 min) was significantly higher than IM group (521 ± 421 min) (Fig. 3c).

Three patients required treatment for pruritus in IM group and 2 in IM + PCEA group. One patient in IM group requested treatment for PONV. The difference among the groups was not statistically significant for morphine induced side effects.

**Fig. 4:**
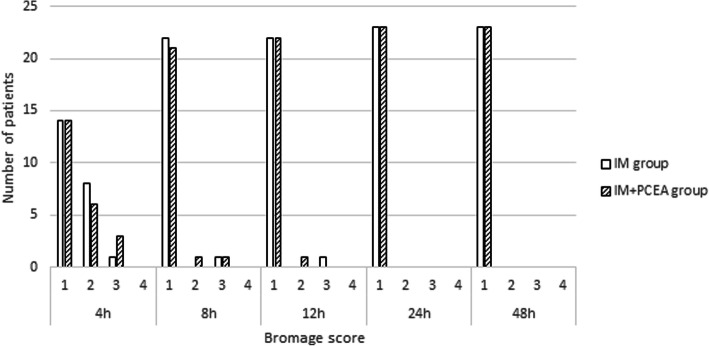
Bromage score of postoperative pain during the first 48 h after intrathecal administration of morphine. Bromage score: score 1 = free movement of legs and feet; score 2 = just able to flex knees with free movement of feet; score 3 = unable to flex knees, but with free movement of feet; and score 4 = unable to move legs or feet. IM = intrathecal morphine. IM + PCEA = intrathecal morphine combined with patient-controlled epidural anesthesia

There were no significant differences in Bromage scores during the first 48 h between two groups (Fig. 4). All patients were evaluated as 1 in Bromage score from 24 h after intrathecal administration of morphine. Ambulation was delayed for approximately 24 h in one patient in IM group due to postoperative pain. Two patients in IM + PCEA group experienced delayed ambulation for approximately 1 and 6 h, respectively, due to weakness of lower extremities. There were no patients who experienced neurological complications or respiratory depression. All patients discharged from the hospital on day 7 after the surgery as scheduled. We obtained patient satisfaction score from 89% of the participants (21/23 in IM group and 20/23 in IM + PCEA group). There was no significant difference in patient satisfaction score between IM group (3.57 ± 1.36) and IM + PCEA group (4.23 ± 0.73) (*p* = 0.0651). Although no patient gave satisfaction score of 1 in IM + PCEA group, three patients in IM group scored 1.

## Discussion

By analyzing 46 patients scheduled for cesarean delivery in this prospective randomized study, we found that the combined use of PCEA and intrathecal morphine provides better post-cesarean section analgesia in the first 12 h at rest and in the first 24 h at movement compared to intrathecal morphine alone. In addition, although no significant difference in patient satisfaction score between IM group and IM + PCEA group was observed, a trend of higher satisfaction was seen in IM + PCEA group. To the best of our knowledge, this is the first study to compare intrathecal morphine with PCEA and intrathecal morphine alone. Similarly to our results, when focusing on the advantages of PCEA, a previous study concluded that the combined use of intrathecal morphine and PCEA improved post-cesarean section analgesia compared to PCEA without opioids [2]. Another study reported that intrathecal morphine alone was superior to epidural morphine alone or PCEA without opioids [1]. Accordingly, PCEA alone is likely to be inferior to intrathecal morphine alone for post-cesarean section analgesia. While the literature showed that intrathecal morphine provided better post-cesarean section analgesia compared to epidural morphine or PCEA without opioids [1], the pain scores during mobilization in the IM group in our study were similar to the present study. Therefore, intrathecal morphine alone may not be the best post-cesarean section analgesia. Moreover, previous studies have demonstrated that the suitable target for optimal analgesia is NRS score of below 3–3.3/10 and reductions in pain scores of 30–40% [9–13]. Therefore, despite the statistical differences, postoperative analgesia at rest seems to be sufficient in both IM group and IM + PCEA group. By contrast, postoperative analgesia during mobilization in IM group seems to be insufficient (mean NRS range 4.8–5.7/10) and additional thoracic PCEA (IM + PCEA group) provided clinically meaningful reduction (reduction to mean NRS range 1.5–3.5/10) in pain scores and optimal analgesia.

High quality post-cesarean section analgesia is crucial for postoperative recovery, as patients are recovering from major abdominal surgery while breastfeeding and caring for a newborn [14, 15]. To provide adequate post-cesarean section analgesia, clinical management guidelines for obstetrician-gynecologists recommends a multimodal approach in which systematic opioids can be reduced [16]. In guidelines of Enhanced Recovery After Surgery (ERAS) Society, thoracic epidural analgesia is an alternative to intrathecal morphine for post-open general gynecologic surgery analgesia [17]. Further, despite the benefit in analgesia, several risks of adding thoracic epidural anesthesia should be taken into consideration. First, patients will receive needle puncture twice to perform lumbar spinal and thoracic epidural anesthesia. Second, use of a combined lumbar epidural and spinal technique will save an additional procedure, however, lumbar epidural analgesia may increase motor blockade which can contribute to delay in ambulation. In the present study, all patients in IM + PCEA group were evaluated as 1 on Bromage score, indicating free movement of legs and feet, with two patients (8.7%) experiencing delayed ambulation. The low incidence of delayed ambulation may be linked to the level of placement of the epidural catheter, which in the present study was the lower thoracic vertebral interspace (Th11–12 or Th12-L1). While motor blockade during thoracic epidural analgesia has been reported to be 6.7% at 24 h after cesarean section [2], the incidence of motor blockade was shown to be 26% at 12 h after cesarean section during continuous epidural infusion via L2–3 or L3–4 vertebral interspace in prior studies [18]. Therefore, lumbar epidural analgesia may increase the risk of motor blockade compared to thoracic epidural analgesia. Furthermore, it is known that adequate postoperative analgesia is necessary for early ambulation in addition to recovery of the motor function [19]. Likewise, one patient (4.3%) in the IM group (who did not receive epidural anesthesia) experienced delayed ambulation for approximately 24 h due to postoperative pain. Third, neurological complications are a rare but serious complication associated with epidural anesthesia. Although there were no patients with neurological complications in our study, previous literature has reported the incidence of permanent neurological injury after epidural anesthesia as 0–7.6:1000 [20].

In addition, the choice and concentration of epidural local anesthetic play an important role in post-cesarean section analgesia and early ambulation. The choice of local anesthetic usually depends on the speed of onset required for the particular clinical situation. Epidural local anesthetics commonly used for cesarean section analgesia include lidocaine, bupivacaine, ropivacaine, and chloroprocaine. Previous studies have used 0.1 to 0.2% ropivacaine or levobupivacaine for PCEA following cesarean section [1, 2, 4, 21]. One study comparing 0.15% of plain ropivacaine and levobupivacaine for post-cesarean section PCEA showed no significant differences regarding postoperative analgesic efficacy and motor weakness [4]. Another study which compared low concentration (0.15%) and high concentration (0.5%) levobupivacaine for postoperative epidural analgesia after major abdominal surgery reported that there were no significant differences in analgesic effect with consistent low motor blockade [22]. Therefore, low concentrated levobupivacaine may be a potential alternative for ropivacaine. In the present study we chose 0.167% levobupivacaine as literature on levobupivacaine for post-cesarean section PCEA was limited. The present study showing low NRS scores without motor weakness in IM + PCEA group supports that low concentrated levobupivacaine may be optimal for post-cesarean section PCEA.

The study has several potential limitations. First, although patients were randomly divided into two groups, PCEA did not allow a full blinding. However, the data was collected by an investigator who was not involved in providing anesthesia. Another method to minimize performance bias is to compare IM + PCEA with levobupivacaine versus IM + PCEA with saline alone as control group, however, we were unable to conduct this for ethical concerns. Second, only elective cesarean sections were included in our study as we were unable to obtain informed consent in extremely emergent cesarean sections. This may limit the generalizability of our inferences to more severe cases. However, our findings remain highly relevant to the majority of the population who receive cesarean sections. Third, scheduled acetaminophen and/or non-steroidal anti-inflammatory drugs (NSAIDs) were not used in our study. Scheduled acetaminophen and/or NSAIDs is a less invasive form of multimodal pain control compared to epidural anesthesia and previously reported to improve post-cesarean section analgesia [23, 24]. If the scheduled acetaminophen and/or NSAIDs were used in this study, it might decrease the impact of PCEA. Forth, patients were not allowed to ambulate with the epidural in place and the thoracic epidural catheter was removed in 24 h after intrathecal administration of morphine. We speculate that a thoracic PCEA might be beneficial in the setting of a longer period of infusion and patients being permitted to ambulate with the epidural catheter in place.

## Conclusions

A combined use of PCEA and spinal anesthesia with intrathecal morphine provided better postoperative analgesia following cesarean section without delay in ambulation compared to single shot spinal anesthesia with intrathecal morphine alone. Although there are several risks that require consideration, thoracic epidural catheterization and 24 h of PCEA in addition to intrathecal morphine may be a reasonable option to improve post-cesarean section analgesia especially during mobilization.

## Data Availability

The datasets used and analyzed during the current study are available from the corresponding author on request.

## Abbreviations

PCEA: Patient-controlled epidural anesthesia
PCA: Patient-controlled anesthesia
NRS: Numerical rating scale
PONV: Postoperative nausea and vomiting
SD: Standard deviation
ERAS: Enhanced Recovery After Surgery
NSAIDs: Non-steroidal anti-inflammatory drugs
